# Biosynthetic Mechanism for Sunscreens of the Biocontrol Agent *Lysobacter enzymogenes*

**DOI:** 10.1371/journal.pone.0066633

**Published:** 2013-06-24

**Authors:** Yan Wang, Guoliang Qian, Yaoyao Li, Yansheng Wang, Yulan Wang, Stephen Wright, Yuezhong Li, Yuemao Shen, Fengquan Liu, Liangcheng Du

**Affiliations:** 1 Department of Chemistry, University of Nebraska-Lincoln, Lincoln, Nebraska, United States of America; 2 Department of Plant Pathology, Nanjing Agricultural University, Nanjing, China; 3 State Key Laboratory of Microbial Technology, College of Life Sciences, Shandong University, Jinan, China; University of Manchester, United Kingdom

## Abstract

*Lysobacter* are ubiquitous environmental bacteria emerging as novel biocontrol agents and new sources of anti-infectives. So far, very little effort has been invested in the study of the biology of these Gram-negative gliding bacteria. Many *Lysobacter* species are characterized by their yellow-orange appearance. Using transposon mutagenesis, we identified a stand-alone polyketide synthase (PKS) gene cluster required for the pigment production in *L. enzymogenes* OH11. The yellow pigments were abolished in the “white” mutants generated by target-specific deletions of ketosynthase (KS), acyl carrier protein, or ketoreductase. Spectroscopic data suggested that the pigments belong to xanthomonadin-like aryl polyenes. Polyene-type polyketides are known to be biosynthesized by modular PKS (Type I), not by stand-alone PKS (Type II) which always contain the heterodimer KS-CLF (chain-length factor) as the key catalytic component. Remarkably, this aryl polyene PKS complex only contains the KS (ORF17), but not the CLF. Instead, a hypothetical protein (ORF16) is located immediately next to ORF17. ORF16–17 homologs are widespread in numerous uncharacterized microbial genomes, in which an ORF17 homolog is always accompanied by an ORF16 homolog. The deletion of ORF16 eliminated pigment production, and homology modeling suggested that ORF16 shares a structural similarity to the N-terminal half of CLF. A point-mutation of glutamine (Q166A) that is the conserved active site of known CLF abolished pigment production. The “white” mutants are significantly more sensitive to UV/visible light radiation or H_2_O_2_ treatment than the wild type. These results unveil the first example of Type II PKS-synthesized polyene pigments and show that the metabolites serve as *Lysobacter* “sunscreens” that are important for the survival of these ubiquitous environmental organisms.

## Introduction

The genus *Lysobacter* is one of the most ubiquitous environmental microorganisms, existing in such diverse habitats as marine thermovents, tar pits, compost sludge and volcanic ash, as well as soil and aquatic environments [1], [2], [3]. Since the classification of the genus by Christensen and Cook in 1978, a large number of lytic enzymes and antibiotic metabolites have been discovered from various *Lysobacter* species [1], [3], [4], [5]. In the recent years, these Gram-negative gliding bacteria have emerged as novel biocontrol agents against pathogens of crop plants. Among them, *L. enzymogenes* is the best studied species [2], [6], [7]. It exhibited field efficacy against diseases of *Bipolaris* leaf spot of turfgrass caused by *Bipolaris sorokiniana* [8], brown patch caused by *Rhizoctonia solani* [6], [9], stem rust caused by *Puccinia graminis* and bean rust caused by *Uromyces appendiculatus* [10]. In greenhouse experiments, it also exhibited efficacy against summer patch of Kentucky bluegrass caused by *Magnaporthe poae* [11] and suppressed damping-off of sugar beet caused by *Pythium ultimum* [12].

In addition, *Lysobacter* are new sources for bioactive natural products [4], [5]. For example, HSAF (dihydromaltophilin) produced by *L. enzymogenes* is a broad spectrum antifungal compound, which has novel structural features and a mode of action distinct from existing fungicides on the market [13], [14]. HSAF is also a critical factor of *L. enzymogenes* as a biocontrol agent against plant fungal diseases [13], [15], [16]. Several *Lysobacter* species produce potent antibacterial metabolites. These include the cyclic peptide lysobactin [17], [18], [19] and tripropeptins [20], [21], the cephem-type β-lactam antibiotic cephabacins [22], [23], [24], and the cyclic lipodepsipeptide WAP-8294A [25], [26], [27], [28]. The WAP-8294A group is particularly noteworthy because of their potent anti-MRSA (methicillin-resistant *Staphylococcus aureus*) activity and the clinical trials of WAP-8294A2 [29].

Despite their great potentials as emerging biocontrol agents and a new source of bioactive compounds, very little effort has been invested in the study of the biology of the bacteria. Traditionally, Gram-positive soil bacteria, particularly the *Streptomyces*, have been the source of bioactive natural products and biocontrol agents. The biology, ecology, and biochemistry of *Streptomyces* have been subject to investigations for over half a century. The insights gained from these investigations have helped the better utilization of the Gram-positive bacteria for drug discovery and development of agrochemicals and animal health products. In contrast, very little is known about the Gram-negative *Lysobacter* species. A characteristic feature of many *Lysobacter* species is the yellow-orange appearance. The nature of the pigments, the genetic basis for their production, and their biological function were not clear. The goal of this study is to identify the chemical nature of the characteristic pigments and to study the biosynthetic mechanism and biological function of these pigments. Our study revealed that the *Lysobacter* yellow pigments are non-carotenoid polyene compounds and their biosynthesis involves an unusual type II polyketide mechanism. These compounds serve as “sunscreens” for photo-survival of these ubiquitous environmental microorganisms.

## Results and Discussion

### The “Yellow” Locus of *Lysobacter* Identified by Transposon Mutagenesis

**Figure 1:**
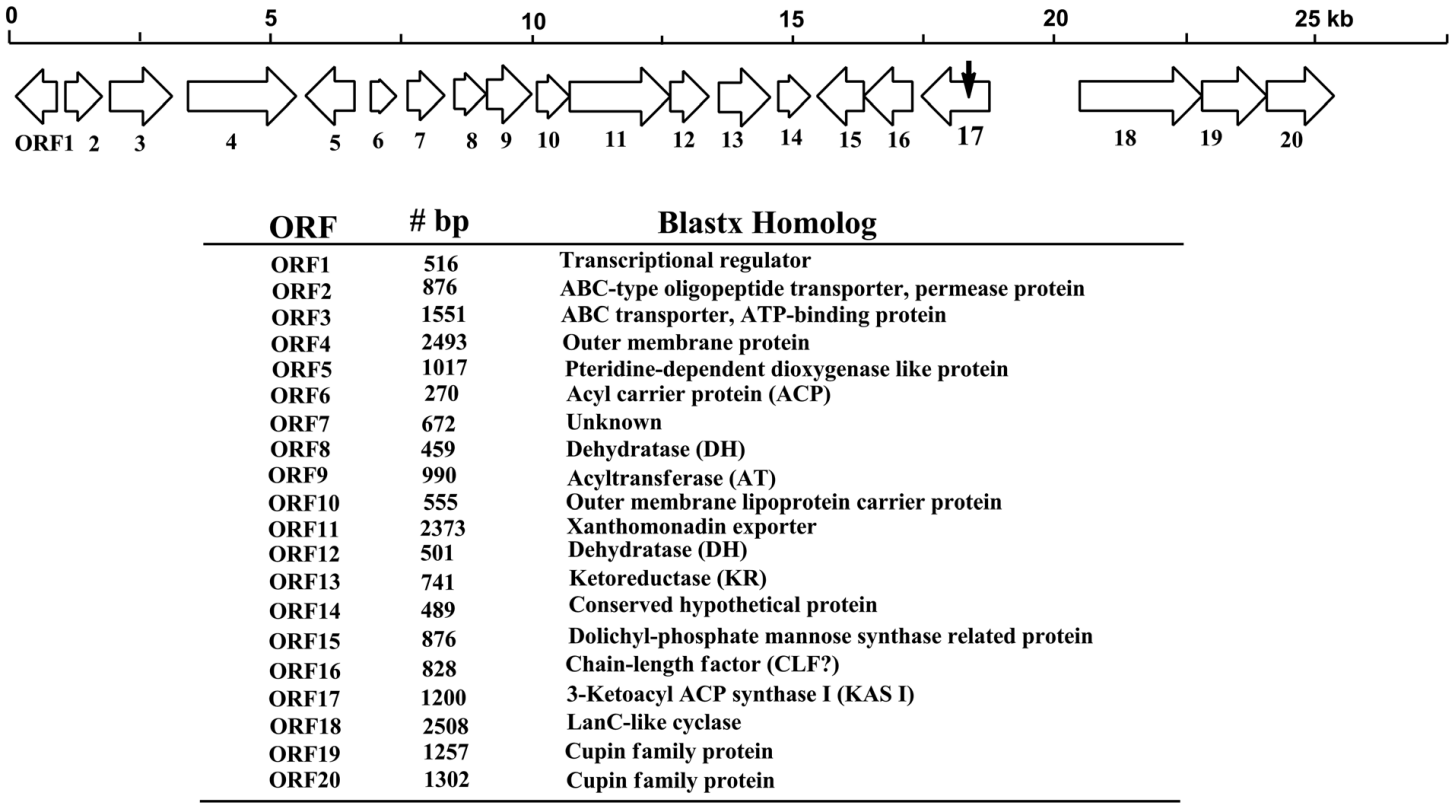
The biosynthetic gene cluster for the yellow pigments of *L. enzymogenes* OH11. The small arrow on ORF17 indicates the transposon insertion site in the initial “white” mutant OH11B.

**Figure 2:**
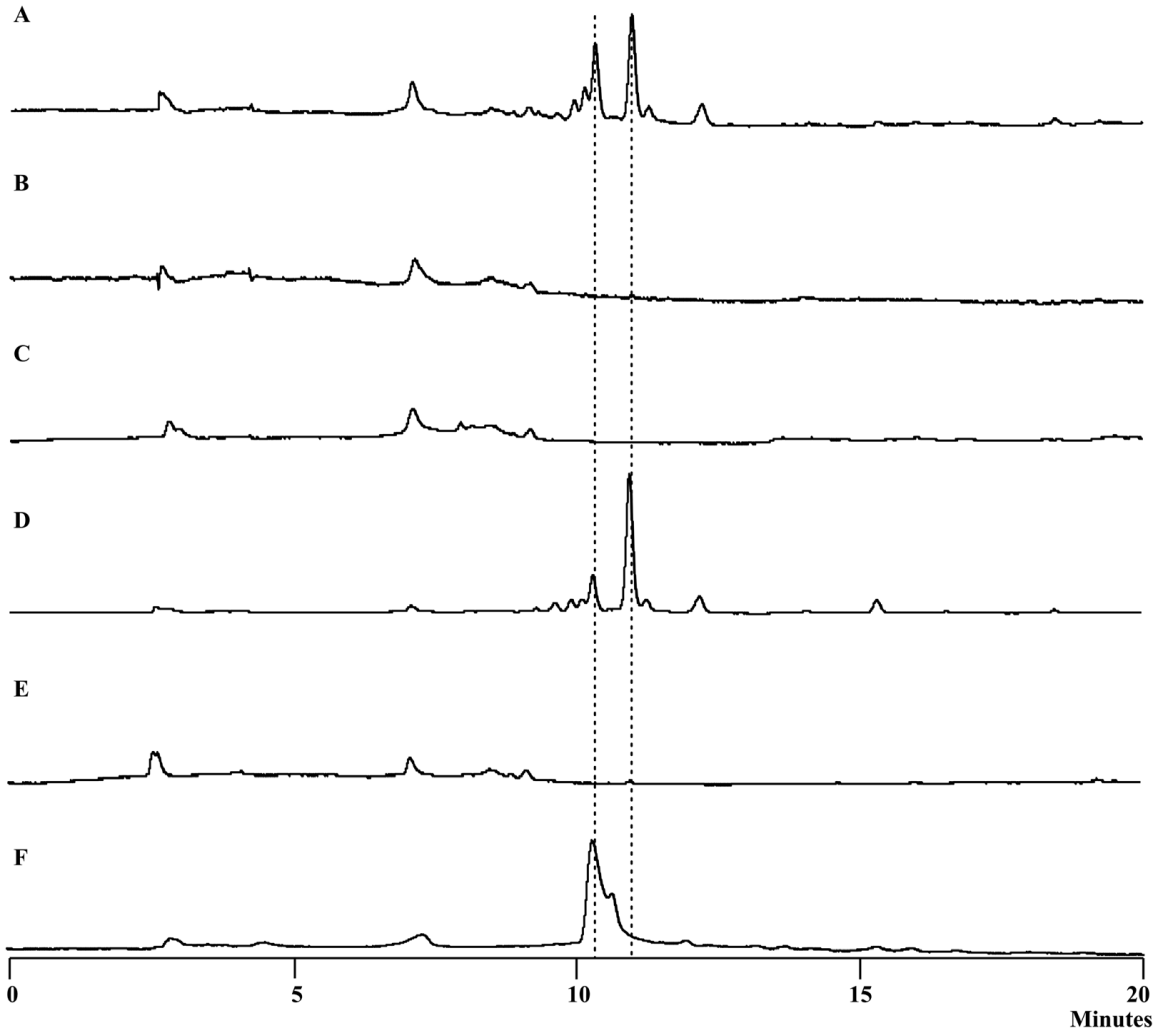
HPLC of the yellow pigments of *L. enzymogenes* OH11. A. wild type; B. ΔORF17 (KAS I); C. ΔORF6 (ACP); D. ΔORF10 (outer membrane lipoprotein carrier protein); E. ΔORF13 (KR); F. xanthomonadin extract from *Xanthomonas campestris* pv. *campestris* (as a reference).

To understand the nature of the characteristic yellow-orange appearance of *Lysobacter* species, we first generated a library of *L. enzymogenes* OH11 mutants by using the *mariner* transposon on pSC137 (*E. coli* SM10λpir as host) [30]. The screening of transconjugants led to the identification of one “white” colony, OH11B (Figure S1 in File S1). The sequencing of the flanking regions of the transposon insertion site revealed a gene (*KasI*) encoding 3-ketoacyl-acyl carrier protein synthase I (KAS I, or ketosynthase) (Figure 1). To verify the role of *KasI* in yellow pigment production, *KasI* gene was target-specifically deleted and the resulting mutants exhibited the expected “white” phenotype. Subsequent HPLC analysis confirmed that the pigments present in the wild type were absent in the *KasI* deletion mutant (Figure 2). This result confirmed that *KasI* is indeed required for the production of the yellow pigments.

### A Type II PKS Gene Cluster Required for the Pigment Biosynthesis

To determine the genes relevant to the pigment production, we analyzed the putative open reading frames (ORF) surrounding *KasI* in the genome sequence of strain OH11 [31]. A total of 20 ORFs were identified at the *KasI* locus, among which 17 ORFs (ORF1 to ORF17) were closely clustered with *KasI* (ORF17, Figure 1). The region downstream to ORF1 contains multiple transporters for phosphate and sulfate, which are likely irrelevant to the production of yellow pigments. No obvious ORF was identified in the 2.2 kb region between ORF17 and ORF18. ORF18, 19 and 20 appear translationally coupled, as the stop codon of one ORF overlaps with the start codon of the next ORF. The transcriptional direction of ORF18, 19 and 20 is opposite to that of ORF17 (*KasI*) (Figure 1). The sequence analysis suggests that the region from ORF1 to ORF17 is likely to constitute the gene cluster required for pigment production. Five types of genes are found in the cluster (Figure 1). The first type is predicted to encode the stand-alone (type II) PKS (polyketide synthase), including ORF17/16, 9, 6, 13, 8 and 12 that encode ketosynthase/hypothetical protein, AT (acyltransferase), ACP (acyl carrier protein), KR (ketoreductase), DH (dehydratase) and DH, respectively. It is interesting that two putative DH are present in the cluster, and both show homology to FabA/FabZ, the DH of fatty acid synthase in *E. coli* [32], [33], [34]. The second type is a putative tailoring gene, ORF5, encoding a pteridine-dependent dioxygenase [35], [36]. The third type is putative transport-related genes, including multiple exporters (ORF2, 3, 11), outer membrane protein (ORF4), outer membrane protein carrier (ORF10), and pigment modifying enzyme for membrane targeting (ORF15). The fourth type is a putative transcriptional regulator gene, ORF1, encoding a protein homologous to the BadM/Rrf2 family [37]. The last type is unknown or hypothetical proteins (ORF7 and 14).

To obtain further evidence for the relevance of this gene cluster, we generated mutants of several selected genes within the cluster (Figure S2 in File S1). ORF1 was chosen because it is likely to be the pathway specific regulator. ORF1 mutant exhibited the yellow phenotype, indicating that ORF1 is not essential for pigment production. Similarly, ORF10 mutant still produced the same pigments as the wild type (Figure 2 and S2 in File S1). An initial Blast search of ORF10 gave hits of acyl-CoA synthetases. However, a protein homology detection and structure prediction using the HHPred iterative server [38] predicted that ORF10 is homologous to outer-membrane lipoprotein carrier proteins. This putative lipoprotein transporter is not essential for pigment production. In contrast, ORF6 mutant and ORF13 mutant were deficient in the yellow pigments (Figure 2 and S2 in File S1). ORF6 and ORF13 encode ACP and KR, respectively, both of which are components of fatty acid synthases (FAS) and polyketide synthases (PKS). Together with the results from ORF17 (KS) mutant, the data indicate that yellow pigment biosynthesis involves a FAS/PKS mechanism. Finally, ORF18 mutant still produced the yellow pigments (Figure S2 in File S1), suggesting that the last three ORFs are not part of the pigment biosynthetic gene cluster. Together, the data verified that a type II PKS complex is responsible for pigment production in *L. enzymogenes*.

### Aryl Polyenes as *Lysobacter* Yellow Pigments

**Figure 3:**
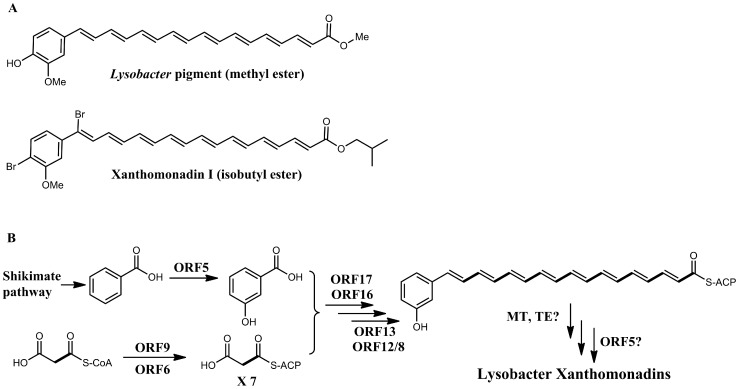
The proposed chemical structure and biosynthetic mechanism for the yellow pigments. A. The proposed chemical structure of the main yellow pigment of *L. enzymogenes* OH11 and xanthomonadin I from *Xanthomonas campestris* pv. *campestris*. B. The proposed biosynthetic pathway for *Lysobacter* pigments. The type II PKS consists of ORF17/16, 9, 6, 13, and 12/8, corresponding to KS/CLF, AT, ACP, KR, and DH/DH, respectively. ORF5 (pteridine-dependent dioxygenase) is proposed to add the 3-hydroxy to benzoic acid to form the starter unit of biosynthesis. An unknown thioesterase (TE) releases the initial aryl octaene intermediate, which is methylated by an unknown methyltransferease (MT) and 4-hydroxylated by a putative hydroxylase (or ORF5).

To determine the chemical structure of the pigments, we prepared the main yellow pigment through multiple steps of extraction and column chromatography. The pigment appeared unstable and, once purified, quickly became insoluble in common organic solvents. Nevertheless, we were able to obtain a mass spectrum of the main pigment using a freshly prepared sample (Figure S3 in File S1). The main signal was a *m/z* 391.4 for [M+H]^+^, with its [M+Na]^+^413.6 and [M+K]^+^429.4. Due to the difficulty in obtaining a sufficient amount of *Lysobacter* pigments for further structural analysis, we extracted the pigments from *Xanthomonas campestris* pv. *campestris*, which also produce yellow pigments known as xanthomonadins [39], [40], [41]. Both *Xanthomonas* and *Lysobacter* belong to the family Xanthomonadaceae within the gamma proteobacteria and exhibit a similar yellow color. HPLC results showed that the pigments from *Lysobacter* have the similar retention times as that from *Xanthomonas* (Figure 2). The maximal visible light absorptions of the *Lysobacter* pigments were 430 and 453 nm in chloroform, which are similar to those for xanthomonadins (Figure S3 in File S1) [41], [42]. The *Xanthomonas* pigments are a mixture of aryl polyenes, differing mainly in the number of methylation and bromination (0–2 Br) (Figure 3) [41], [42]. A halogenase gene is present in the xanthomonadin gene clusters found in two *Xanthomonas* species [35], [43]. In contrast, the *Lysobacter* gene cluster does not contain a halogenase gene (Figure 1). The results suggest that the *Lysobacter* pigments are non-brominated analogs of xanthomonadins. Consistent with this proposal, the mass spectrum of the *Lysobacter* pigment did not show the characteristic isotopic ions of brominated compounds (Figure S3 in File S1).

**Figure 4:**
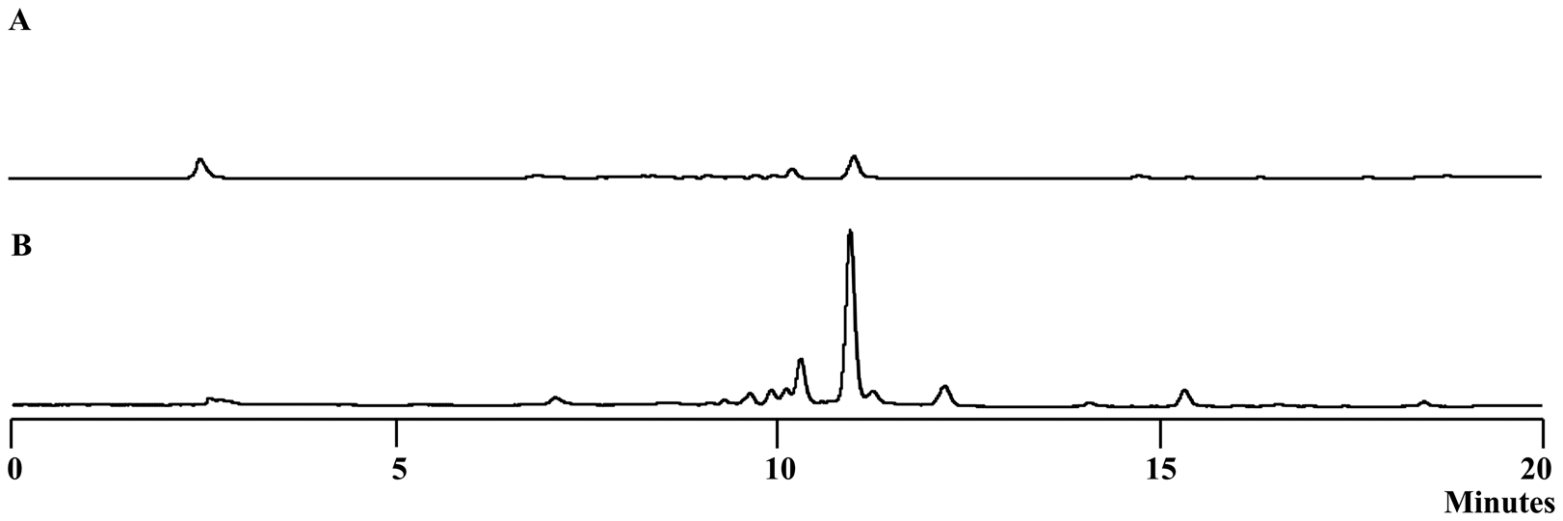
HPLC of the yellow pigments of *L. enzymogenes* OH11. A. ΔORF5 (Pteridine-dependent dioxygenase); B. ΔORF5 fed with 3-hydroxybenzoic acid.

To obtain more information about the nature of the *Lysobacter* pigments, we generated a deletion mutant of ORF5, which encodes a pteridine-dependent dioxygenase (Figure 1). An ORF5 homolog (*xanB2*) is also present in the xanthomonadin gene clusters in *Xanthomonas. XanB2* is required for the formation of 3-hydroxybenzoic acid (3-HBA), which is likely the precursor of xanthomonadin biosynthesis [35], [36]. The deletion of ORF5 resulted in ∼90% reduction of pigment production in *L. enzymogenes* (Figure 4). When 3-HBA was externally added into the culture of ORF5 mutant, the pigment production was fully restored. This result supports that the *Lysobacter* pigments are analogs of xanthomonadins of *X. campestris*, which uses 3-HBA as a precursor for the pigment biosynthesis. Based on these results, we propose that the chemical structure of the main *Lysobacter* pigment is an analog of xanthomonadins, probably an aryl octaene (Figure 3). A number of xanthomonadins were reported from various *Xanthomonas*. However, xanthomonadin I, a dibrominated aryl octaene (isolated as isobutyl ester, Figure 3), is the only one with an established structure (by X-ray crystallography) [41]. These compounds are highly unstable, probably due to the photo reactive nature of the fully conjugated polyenes.

### Unusual KS-CLF in the Polyene Type II PKS

**Figure 5:**
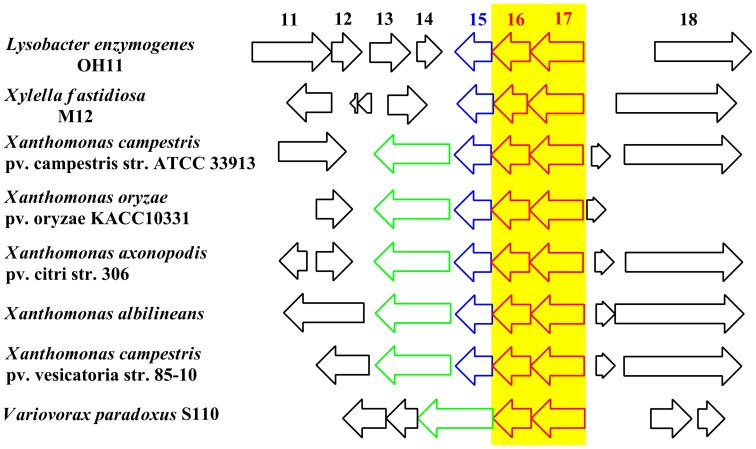
The highly conserved ORF16–ORF17 pair from a number of selected examples of microbial genomes. For each of the biosynthetic gene clusters, only part of the cluster is shown. The organization of the ORFs flanking ORF16–ORF17 is not conserved, except the blue-colored ORF15 homologs (dolichyl-phosphate mannose synthase, absent in *Variovorax paradoxus*) and the green-colored halogenase genes (absent in *L. enzymogenes* and *Xylella fastidiosa*).

**Figure 6:**
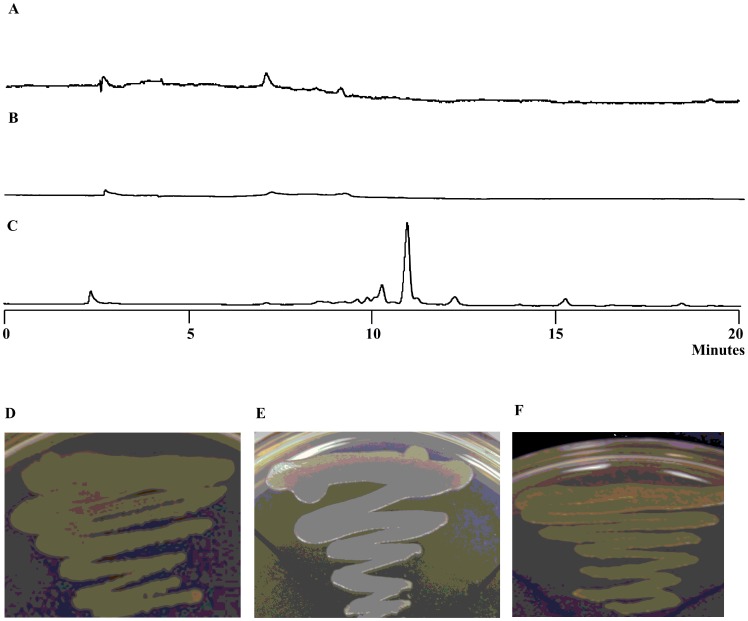
HPLC of the yellow pigment production in three ORF16 mutants of *L. enzymogenes* OH11. A. ΔORF16; B. ORF16 Q166A; C. ORF16 S120A. The phenotype of the wild type (D), ORF16 Q166A (E), and ORF16 S120A (F) is also shown.

Polyene-type polyketides are typically biosynthesized by modular PKS (Type I), such as the PKSs for antifungal antibiotics amphotericin B, nystatin, and pimaricin (natamycin) [44], [45], [46], [47] and the yellow pigment DKxanthenes that are important to fruiting body formation and sporulation in *Myxococcus xanthus* [48]. A type II PKS for polyene biosynthesis is unusual, and this made us look into the components of the PKS complex, which is known to contain a ketosynthase/chain length factor (KS-CLF, also known as KS_α_-KS_β_) heterodimer as the essential component. The role of the heterodimer in type II polyketide chain initiation and elongation has been well documented in the literature [49], [50], [51], [52]. However, the PKS complex for *Lysobacter* pigments only contains a homolog of KS (ORF17, containing the highly conserved **C**SSS motif, with cysteine as the active site residue), but no CLF. The KS is immediately followed by a hypothetical protein (ORF16) (Figure 1). Blast search of ORF16 did not show any obvious function. Interestingly, homologs of the ORF16–17 pair are widespread in numerous genomes of microbes, such as *Xanthomonas*, *Xylella*, and *Variovorax* (Figure 5). Although the organization of these gene clusters varies significantly, they all share a common feature, which is that the homolog of ORF17 is always accompanied by the homolog of ORF16 (Figure 5). To obtain clues for the function of this hypothetical protein in pigment production, we generated an ORF16 deletion mutant. The mutant did not produce the pigments (Figure 6), showing the essential role of this hypothetical protein. Subsequently, we conducted a structure-based HHPrep search for ORF16. Although ORF16 does not appear homologous to any known protein, the search showed that ORF16 shares a structural similarity to the N-terminal half of CLF (Figure S4 in File S1). The homology includes several conserved structural elements, such as the α3 helix that, with KS, is involved in the “polyketide tunnel” formation, the loop between α3 and α4 helices, and the α6 helix that contains a conserved residue glutamine [52]. The glutamine in the α6 helix is related to the decarboxylase function of CLF during the initiation of type II polyketide biosynthesis [51]. All ORF16 homologs share a conserved glutamine (Q166 of *Lysobacter* ORF16) in the α6 helix at a region near the conserved glutamine of tetracenomycin CLF (Tcm_CLF, M80674) and actinorhodin CLF (Act_CLF, X63449) (Figure S4 in File S1) [53], [54]. An ORF16 mutant with a point-mutation at the glutamine (Q166A) was subsequently generated. In addition, a mutant S120A was generated because this serine residue is in the loop between α3 and α4 helices and is conserved in all ORF16 homologs, as well as in Tcm_CLF, Act_CLF and Act_KS (Figure S4 in File S1). While mutant S120A exhibited the wild type phenotype and produced the pigments, mutant Q166A showed the pigment deficient phenotype and produced no pigment (Figure 6).

To our knowledge, this is the first example that a polyene-type polyketide is biosynthesized by type II PKS. The type II PKS mechanism had been proposed for the biosynthesis of xanthomondins from *X. oryzae* pv. oryzae, although several key components (KS, CLF and KR) were not identified in the sequenced region [43]. Our results indicate that the hypothetical protein (ORF16) may be a novel type of CLF. Based on the data obtained in this study and general paradigm of type II PKS-catalyzed biosynthesis, we propose a biosynthetic pathway for *Lysobacter* xanthomonadin-like pigments (Figure 3). The biosynthesis is initiated by a 3-hydroxylation of benzoic acid, which is catalyzed by pteridine-dependent dioxygenase (ORF5) [35], [36]. The resulted starter unit, 3-hydroxybenzoic acid, is condensed with a decarboxylated malonate, which is carried out by KS/CLF, AT, and ACP of the type II PKS system. The KR then reduces the β-keto to β-hydroxy, which is then dehydrated by the DH to generate the double bonds. Two DH are present in this PKS system, resembling FabA and FabZ in fatty acid synthesis [32], [33], [34]. The specificities of the two DH in pigment biosynthesis require further biochemical characterization. A number of tailoring enzymes that are required for polyketide modifications are missing in the gene cluster. These include an enzyme, such as a thioesterase (TE), for the release of the initial aryl octaene intermediate, a methyltransferease (MT) for O-methylation of 3-hydroxy group, and a hydroxylase for 4-hydroxylation of the benzene ring. The pigment biosynthetic pathway may recruit enzymes from other pathways for these modification steps, although the 4-hydroxylation of the benzene ring could also be carried out by ORF5.

### Protection from Damage by UV-visible Light and H_2_O_2_

**Figure 7:**
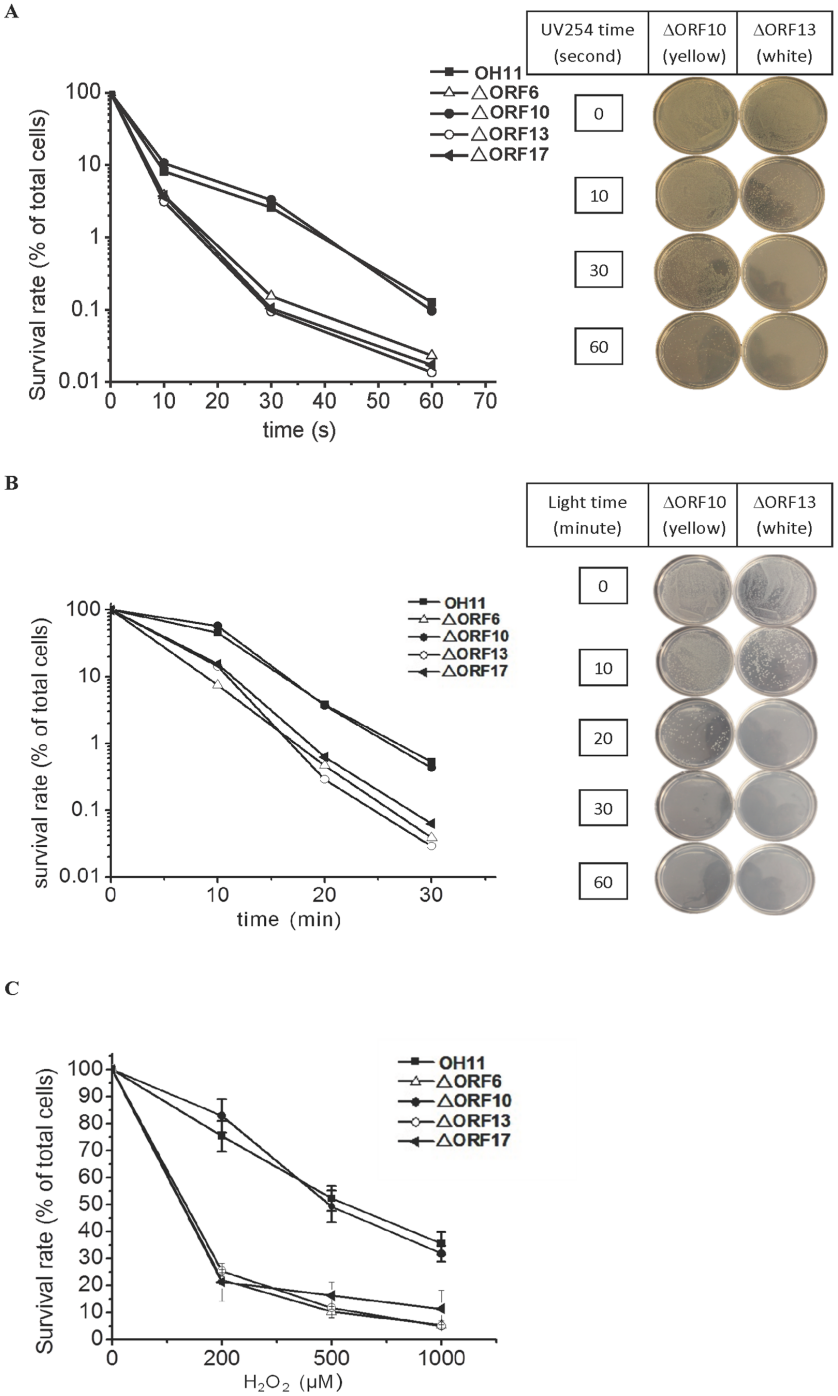
The survival rate of the wild type (OH11) and four mutants upon exposure to UV254 (A), visible light (B), or H_2_O_2_ (C). In A and B, each data point represents the average of two replicates; in C, each data point is of three replicates with deviation bars shown.

To determine the biological function of the pigments, the wild type strain and the mutant strains were radiated with UV 254 nm or visible light (Figure 7). The pigment deficient strains were significantly more sensitive to the radiation than strains with the yellow pigments. For example, under UV 254 nm, approximately 0.1–0.2% cells of the pigment deficient strains (mutants of ORF6, 13, and 17) survived 30 seconds of radiation, while 4–5% cells of the strains with yellow phenotype (wild type and ORF10 mutant) were still alive under the same condition. Under visible light, approximately 0.3–0.6% cells of the pigment deficient strains survived radiation for 20 minutes, while 4% cells of the strains with yellow phenotype remained alive under the same condition. The same trend was observed when the cells were treated with H_2_O_2_ (Figure 7). The pigment deficient strains had a survival rate of approximately 25% under 200 µM H_2_O_2_ treatment, while the strains with yellow phenotype had a survival rate of approximately 80% at the same condition.

The gliding Gram-negative bacteria *Lysobacter* are emerging as effective biocontrol agents for crop diseases and new sources for bioactive natural products. Several reports had examined the biocontrol efficacy of *Lysobacter* species and their colonization in soil and roots/rhizosphere, and recent research on *Lysobacter* has focused on the identification and utilization of the bioactive molecules [5], [13], [15], [19], [28], [31], [55], [56], [57], [58], [59]. Relatively little has been carried out to investigate the biology of these ubiquitous microorganisms. Many *Lysobacter* species appear in yellow-orange color for the colonies and cultures. In this study, we investigated the chemical structure, the biosynthetic mechanism, and the biological function of the yellow pigments in *L. enzymogenes* OH11. The results showed that pigments are xanthomonadin-like aryl polyene metabolites. To our knowledge, this is the first time that xanthomonadin-type of pigments have been identified in a microorganism outside the genus *Xanthomonas*. These metabolites were used as chemotaxonomic markers for *Xanthomonas* species, as pigments of different species appear to have distinct patterns of bromination and methylation on the aryl polyene skeleton [40], [41].

Our results provide evidence for the first characterized example of a type II PKS-catalyzed aryl polyene biosynthesis, which was previously known to require a modular type I PKS. Most intriguingly, the PKS complex contains an unusual KS/CLF that is present in numerous uncharacterized gene clusters found in microbial genomes in databases. These results lay the foundation for biochemical and structural elucidation of this unusual KS/CLF that may lead to new insights into the molecular mechanism for polyketide chain determination.

Residing in the same cluster along with the type II PKS genes are an unusually large number of transport-related genes. These genes are apparently related to the outer membrane localization of the pigments [43]. *L. enzymogenes* OH11 was originally isolated from the rhizosphere soil of pepper plants [7], while the same species, *L. enzymogenes* C3, was also isolated from grass leaf surface [6]. *Lysobacter* species are ubiquitous environmental microorganisms that are prone to photo-oxidative damage. The outer membrane localization could be crucial for the pigments to execute their photo-protective function. Indeed, the “white” mutants exhibit significantly more sensitive to UV and visible light radiation and H_2_O_2_ treatment than the wild type or “yellow” mutants. For the biocontrol application of *Lysobacter* species, epiphytic survival is essential for the bacteria to effectively execute the protection of crop plants against pathogens [60], [61].

## Materials and Methods

### Strains, Plasmids and Primers

*Lysobacter enzymogens* strains were routinely grown on Luria-Bertani (LB) agar or 1/10-strength tryptic soy broth (1/10 TSB, Sigma) at 30°C. *Escherichia coli* DH5α or *E. coli* Top10 were grown in LB at 37°C and used as the host for general DNA propagations. When required, antibiotics were used at the following concentrations: ampicillin, 100 µg/mL; gentamicin (Gm), 15 µg/mL for *E. coli*/pBBR1-MCS5, 200 µg/mL for *L. enzymogenes*; chloramphenicol (Cm), 100 µg/mL; streptomycin (Sm), 100 µg/mL. Genomic DNA of *L. enzymogenes* was prepared as previously described [12]. Plasmids pGEM-zf series from Promega (Madison, WI) and pANT841 [62] were used for cloning and sequencing. Kits for plasmid preparation and DNA gel extraction were purchased from Qiagen (Valencia, CA). The primers used in this study are described in Table S1 in File S1. DNA manipulations were performed according to standard methods [63].

### Transposon Mutagenesis and Isolation of Pigment Mutants

Random transposon mutagenesis of a streptomycin-resistant (Sm^R^) *L. enzymogenes* strain OH11 was performed by conjugating *E. coli* SM10λpir (pSC137, containing a *mariner* transposon) to create a library of insertional mutants [30]. Transconjugants were selected on LB plates supplemented with 100 µg/mL Sm and 100 µg/mL Cm, at 30°C for 72 h. Individual colonies were picked up and streaked on new plates to verify their resistance to Cm. Preliminary pigment mutants were selected according to the color change of the individual colonies. One pigment mutant, designated as strain OH11B, which was deficient in yellow pigment production on LB agar was selected for further study.

### Verification of the *KasI* Locus for Pigment Production

In order to verify the role of the *KasI* locus, *KasI* gene was specifically inactivated by replacing an internal portion of *KasI* with the Cm resistant gene. Two 600-bp DNA fragments, corresponding to the 5′- and 3′-end of *KasI*, was amplified from strain OH11 genomic DNA using primers OPAC1/OPAC2 and OPAC3/OPAC4, respectively. The DNA fragment obtained by primers OPAC1/OPAC2 was digested with *Eco*RI/*Hin*dIII, and the DNA fragment obtained by primers OPAC3/OPAC4 was digested with *Hin*dIII/*Xba*I. The fragments were then cloned into pUC19 at *Eco*RI/*Xba*I sites, resulting in the construct of pUCOPAC1/4. Next, a *Hin*dIII-digested Cm cassette, which was amplified using primers Cm-1/Cm-2, was cloned into the *Hin*dIII-digested pMDOPAC1/4, resulting in pUCOPAC1/4CM. The plasmid pMDOPAC1/4CM was digested with *Eco*RI/*Xba*I, and the released 2100-bp fragment (OPCA1/2+ Cm cassette+OPCA1/4) was cloned into pEX18GM at the *Xba*I/*Pst*I sites, which produced the construct pEX18GMOPAC1/4CM. Finally, pEX18GMOPAC1/4CM was introduced *E. coli* SM10λpir, and the resulting strain was mated with strain *L. enzymogenes* OH11 to transfer the plasmid by conjugation. Colonies of strain OH11 that were grown on LB agar supplemented with Sm (100 µg/mL) and Gm (200 µg/mL) represented single crossover mutants that contained pEX18GMOPAC1/4CM in the chromosomes. Individual colonies were selected and grown in 10% TSB broth overnight at 30°C with shaking. The cultures were diluted, at 1∶1000 ratio, into fresh 10% TSB broth supplemented with 10% sucrose. After 6 h of incubation, cultures were plated onto 10% TSA supplemented with 10% sucrose and incubated at 30°C for 48 h. Individual colonies were selected and re-streaked on fresh plates to obtain single colonies several times before replicated onto 10% TSA containing gentamicin. The colonies exhibited gentamicin sensitivity and sucrose resistance were the putative double crossover mutants, which were verified by phenotype analysis (deficiency of yellow pigments) and diagnostic PCR using primers Cm-1/Cm-2. The true positive *KasI* deletion mutants were selected for further study, which revealed that the *KasI* gene is one of the 20 open reading frames (ORFs) that are clustered in this locus (*KasI* is ORF17, see Figure 1).

### Identification of the “Yellow Locus” in the Pigment Deficient Mutant

The DNA sequences flanking the transposon in strain OH11B were subcloned using the following procedure. The genomic DNA of strain OH11B was digested by *Eco*RI, and the digested DNA was purified and redissolved in water (10 µL). The genomic DNA was ligated into *Eco*RI-digested pUC19, which was transformed into *E. coli* DH5α. The transformants were selected on the LB agar supplied with Cm (20 µL/mL), and the positive clones were grown in LB containing Cm. The individual plasmids were prepared from the clones and digested with *Eco*RI to confirm the inserted DNA fragments. Finally, the inserted DNA sequences in the plasmids were sequenced. The plasmid verified for the pigment deficiency was named pUCKASI. To identify the transposon insertion sites, a basic local alignment search tool (BLAST) was used to compare the genome sequence with the plasmid sequence. The transposon insertion site in strain OH11-B was identified as *KasI* gene, which encodes a 3-ketoacyl-acyl carrier protein synthase I.

### Generate Gene Deletion Mutants to Identify the Pigment Biosynthetic Genes

To identify the genes within the *KasI* locus that are required for yellow pigment production, a series of gene deletion mutants were generated. Two homologous fragments were amplified from each of a selected group of ORFs that are clustered in the *KasI* locus. ORF1, 6, 10, 13, and 18 were selected to cover the entire cluster. The primer pairs used for each of the ORFs are listed Table S1 in File S1. Genomic DNA from the wild type *L. enzymogenes* OH11 served as the PCR template.

To construct the deletion vector for ORF1 (516 bp, a putative transcriptional regulator), primers ORF1F1 and ORF1R1 were used to amplify a 300-bp upstream homologous arm, and primers ORF1F2 and ORF1R2 were used to amplify a 500-bp downstream homologous arm. The upstream arm was digested with *Bam*HI/*Bgl*II, and the downstream arm was digested with *Bgl*II/*Hin*dIII. The fragments were then cloned into the conjugal vector pEX18Gm at the *Bam*HI/*Hin*dIII sites. The construct was first transformed into *E. coli* Top10, and the plasmid was prepared and verified by enzymatic digestion. The plasmid was then introduced into the conjugal strain *E. coli* BW20676 and verified by enzymatic digestion and DNA sequencing. Finally, this BW20676 strain was used to mate with *L. enzymogenes* OH11 to obtain gene deletion mutants. The procedure for screening single crossover mutants and the subsequent double crossover mutants was identical to that described above. The double crossover mutant resulting from this ORF1 construct will have a 429-bp deletion within ORF1.

A similar procedure was used to generate mutants for ORF6 (270 bp, putative acyl carrier protein, upstream arm 291 bp, downstream arm 500 bp, leading to a 225 bp deletion within ORF6), ORF10 (555 bp, putative acyl-CoA synthetase, upstream arm 325 bp, downstream arm 485 bp, leading to a 354 bp within ORF10), ORF13 (741 bp, putative ketoreductase, upstream arm 397 bp, downstream arm 519 bp, leading to a 696 deletion within ORF13), ORF18 (2508 bp, putative LanC-like protein, upstream arm 510 bp, downstream arm 873 bp, leading to a 2397 bp deletion within ORF18), and ORF5 (1017 bp, a pteridine-dependent dioxygenase like protein, upstream arm 501 bp, downstream arm 500 bp, leading to a 828 bp deletion within ORF5).

### Generation of Single Amino Acid-mutations in ORF16 and ORF16-disrupted Mutants

The single amino acid-mutants, Q166A and S120A, of ORF16 were generated to obtain clues of the nature of this hypothetical protein in yellow pigment production. For the Q to A mutation, the primers Q/S-up-forw and Q-up-rev were used to amplify the upstream overlapping fragment, and the primers Q-down-forw and Q/S-down-rev were used to amplify the downstream overlapping fragment (Table S1 in File S1 for primers sequences). Genomic DNA from *L. enzymogenes* OH11 served as the template for amplification. After obtaining the two overlapping fragments, an overlapping PCR was carried out using the two fragments as templates and Q/S-up-forw and Q/S-down-rev as primers. The resulting DNA fragment (fragments Q) was then digested with *Xho*I and *Bam*HI and cloned into the conjugal vector pJQ200SK at the *Xho*I/*Bam*HI sites, which produced pJQ200SK-Q. The Q to A change in the construct was verified by DNA sequencing. For the S to A mutation, the method was identical, except that the primers Q/S-up-forw and S-up-rev were used to obtain the upstream overlapping fragment and primers S-down-forw and Q/S-down-rev were used to obtain the downstream overlapping fragment. The final fragment (fragment S) was also digested by *Xho*I and *Bam*HI and cloned into pJQ200SK, which produced pJQ200SK-S. The S to A change in the construct was verified by DNA sequencing. Due to a very low efficiency of double crossover at the single site mutations using the pJQ200SK-based constructs, fragments Q and S were transferred from pJQ200SK vector to pEX18 vector by *Pst*I/*Bam*HI digestion, which produced pEX18-Q and pEX18-S, respectively. In addition, fragment Q was used to generate an ORF16-disrupted mutant. Fragment Q was digested with *Pst*I and *Bam*HI and cloned into pJQ200SK at *Pst*I/*Bam*HI sites, which produced pJQ200SK-ORF16. The procedure for conjugal transfer and mutant screening was identical to that described above. ORF16-disruption mutant was confirmed by a diagnostic PCR using the primers ORF16-V-F and ORF16-V-R to obtain a 936-bp fragment. The final Q166A and S120A changes in ORF16 were confirmed by DNA sequencing of the PCR products amplified from candidate *L. enzymogenes* mutants resulting from a double crossover.

### Isolation and Analysis of the Pigments

*L. enzymogenes* wild type strain OH11 and various mutants were grown in 3 ml 1/10-strength tryptic soy broth (1/10 TSB; Sigma) at 28°C with shaking at 200 rpm overnight. Aliquots of 200 µl cells were transferred to 20 ml fresh 1/10 TSB medium and cultured for another 3 days under the same condition. The cells were collected by centrifugation at 3600 rpm for 30 min and were resuspended in 1 ml methanol. The suspension was shaken at 120 rpm for 3 hours at room temperature. A 100 µl aliquot of each extracts was analyzed by HPLC (ProStar 210, Varian) using a reversed-phase column (Alltima C18LL, 5 μ, 250 mm×4.6 mm). Water/0.025% TFA (solvent A) and acetonitrile/0.025% TFA (solvent B) were used as the mobile phases with a flow rate of 1.0 ml/min. The HPLC program was as follows: a gradient of 5% to 60% solvent B in solvent A in the first 5 min, 60% to 100% B from 5 to 20 min, 100% B from 20 to 23 min, and 100% to 5% B from 23 to 28min. The pigments were detected at 430 nm on a UV-visible detector (ProStar, model 310; Varian). UV-vis absorption spectra of the pigment extractions were recorded on a spectrophotometer (UV-2401PC). For the preparation of standard xanthomonadins, the published protocol was followed [40], [41]. Briefly, *X. campestris* pv. *campestris* was cultured in NYGB for 16 hours at 30 °C to OD_600 nm_ of 1.2. The culture was collected and resuspended in methanol. The solution was boiled for 5 min and kept at room temperature for 30 min. Aliquots of 100 µl were used for HPLC analysis.

### Preparation and Methyl Esterification of the Pigments

The cells (∼120 g) collected from a 5-liter culture were extracted with methanol (3×240 ml), and the methanol extracts were combined and dried. The residues were extracted twice with ethyl acetate/water (1∶1, vol), and the ethyl acetate phase was collected and dried. The resulting residue was extracted with petroleum ether/methanol (1∶1, vol), and the methanol phase was collected and dried. The residues were then extracted with ethyl acetate/water (1∶1, vol), and the ethyl acetate phase was collected and dried. This ethyl acetate extract (1.2 g) was dissolved in methanol and loaded onto a RP-18 column (80 g). The column was washed with 500 ml of water, followed by 200 ml of 30% methanol, 500 ml of 50% methanol, 500 ml of 70% methanol, 500 ml of 100% methanol, and 2.0 liter of 100% methanol. The final methanol elution (2.0 liters), which contained the yellow pigments, was dried to obtain the yellow pigments (∼ 7 mg). To convert the pigments to their methyl esters, the yellow residues were dissolved in 0.35 ml chloroform and 0.1 ml anhydrous methanol, followed by adding 0.05 ml sodium methoxide (0.3 g/ml). The solution was shaken in the dark at room temperature for 4 hours. The reaction was stopped by adding 15 ml ddH_2_O, and the pH was adjusted to neutral by adding 1% dilute hydrochloric acid. The products were extracted with 20 ml chloroform until no color remained in the aqueous phase. The chloroform phase was dried completely, and the yellow residue was re-dissolved in chloroform, which was loaded to a Sephadex LH-20 column (15 g). The column was developed with chloroform/methanol (2∶1, vol), and the fractions containing the yellow color were combined and dried. The residue was briefly washed with a small amount of petroleum ether, and remaining residue (below 1 mg), which contained the pigments, was dissolved in chloroform and subjected to spectroscopic analysis.

### Photosensitivity Test of Mutants

*L. enzymogenes* OH11 and the pigment mutants were grown in 3 ml 1/10 TSB overnight. Each of the cell suspensions (2 ml medium, adjusted to OD_600 nm_ 1.0) was spread on a plate (5.5 cm, inner diameter) and tested for the sensitivity to UV light using the previously described method with some modifications [36]. A 254 nm UV light source (Model ENF-280C, 115 Volts, 60 Hz, 0.20 AMPS) was used, which was placed directly above the plates, at a distance of 2 cm between the light and the cells. After various exposure times, the cell suspensions were serially diluted and spread on fresh 1/10 TSA plates. The numbers of colonies on each plate were counted after 2 days of incubation. For sensitivity to the visible light, the strains were cultured and tested in the presence of toluidine blue, an exogenous photosensitizer that has an maximum absorption at 626 nm, following the method described previously [61]. Each of the cell preparations (2 ml, OD_600 nm_ 1.0) was washed one time in 0.01 M PBS buffer and re-suspended in 6 ml PBS buffer. The cell suspensions were transferred onto individual plates (5.5 cm, inner diameter), and toluidine blue was added to a final concentration of 5 µM. The plates were placed under a 200 W lamp, at a distance of 30 cm between the lamp and the cells, and shaken at 60 rpm at room temperature. After a certain exposure time, the cells were serially diluted and spread on fresh 1/10 TSA plates. Bacterial colonies on each of the plates were counted after 2 days of incubation.

### H_2_O_2_ Sensitivity Assay

*L. enzymogenes* OH11 and various pigment mutants were grown in 1/10 TSB medium overnight. The cultures were then diluted with fresh 1/10 TSB medium to a final OD_600 nm_ of 0.1. The cell suspensions were mixed with H_2_O_2_ at the final concentration of 200, 500, 1000 µM and allowed to incubate at room temperature for 1 hour. The cells were collected and washed with fresh 1/10 TSB medium. Finally, the cells were serially diluted and spread onto 1/10 TSA plates, and the numbers of colonies were counted from each of the plates after 3 days of incubation [36].

## Supporting Information

File S1 Table S1: Primers used in this study. Figure S1: Identification of the yellow pigment deficient *L. enzymogenes* OH11 mutant through transposon random mutagenesis. A. phenotype of colonies. OH11, the wild type; OH11B, the “white” mutant. B. Cloning the flanking region of the transposon insertion site in the mutant. The genomic DNA was digested by *Eco*RI and cloned into pUC19. The colonies with chloramphenicol (Cm) resistance were the putative positive hits, which were verified by DNA sequencing. pUCKASI, *Eco*RI-digested plasmid from Cm-resistant colonies; pUC19-E, *Eco*RI-digested vector pUC19; OH11B-E, *Eco*RI-digested genomic DNA of mutant OH11B. Figure S2: PCR verification of gene deletion mutants and the phenotype of the mutants. A. ΔORF1; B. ΔORF6; C. ΔORF9; D. ΔORF10; E. ΔORF13; F. ΔORF18. M, size markers; lane-1, wild type; the rest lanes, individual colonies that were selected for analysis (the larger size band indicates the wild type phenotype, and the smaller size band the mutants with gene deletions). Figure S3: Mass spectrum of the main yellow pigment of *L. enzymogenes* OH11 and light absorption spectra (400-500 nm) of the pigments extracted from various strains. A. wild type OH11; B. ΔORF17; C. ΔORF6; D. ΔORF10; E. ΔORF13; F. xanthomonadins from *Xanthomonas campestris* pv. *campestris*. Figure S4: Sequence analysis of ORF16. A. Superposition between ORF16 (white, 275 residues) and the N-terminal region of chain-length factor of actinorhodin PKS, Act_CLF (purple, 415 residues, pdb 1TQY, structure of the KS/CLF heterodimer) [52]. The three green boxes indicate the location of the α3 helix, the loop between α3 and α4 helices, and the α6 helix. The serine residue (S120 for *L. enzymogenes* ORF16 and S128 for Act_CLF) that is conserved in all sequences is located in the loop between α3 and α4 helices, and the glutamine residue (Q166 for *L. enzymogenes* ORF16 and Q161 for Act_CLF) that is conserved in all ORF16 homologs is located in the α6 helix. B. Multiple sequence alignment of selected ORF16 homologs found in GenBank. The alignment was carried out by using Clustal 2.1, and only the sequences around the known active site of known CLF and KS are shown. Tcm_CLF, chain-length factor of tetracenomycin PKS (M80674); Act_CLF and Act_KS, chain-length factor and ketosynthase of actinorhodin PKS (X63449). The glutamine residue conserved in the known CLFs is indicated with a red box, and the active site motif, including the absolutely conserved cysteine residue, of the known KS is underlined [51]. Also indicated by boxes are Q166 and S120, which were subject to mutagenesis in ORF16.
(DOC)
