# Supplementary material for: Biosynthetic Mechanism for Sunscreens of the Biocontrol Agent Lysobacter enzymogenes
Source: PLoS One. 2013 Jun 24;8(6):e66633. doi: 10.1371/journal.pone.0066633 (PMC3691225; doi:10.1371/journal.pone.0066633)
Supplement: File S1 — Table S1: Primers used in this study. Figure S1: Identification of the yellow pigment deficient L. enzymogenes OH11 mutant through transposon random mutagenesis. A. phenotype of colonies. OH11, the wild type; OH11B, the “white” mutant. B. Cloning the flanking region of the transposon insertion site in the mutant. The genomic DNA was digested by EcoRI and cloned into pUC19. The colonies with chloramphenicol (Cm) resistance were the putative positive hits, which were verified by DNA sequencing. pUCKASI, EcoRI-digested plasmid from Cm-resistant colonies; pUC19-E, EcoRI-digested vector pUC19; OH11B-E, EcoRI-digested genomic DNA of mutant OH11B. Figure S2: PCR verification of gene deletion mutants and the phenotype of the mutants. A. ΔORF1; B. ΔORF6; C. ΔORF9; D. ΔORF10; E. ΔORF13; F. ΔORF18. M, size markers; lane-1, wild type; the rest lanes, individual colonies that were selected for analysis (the larger size band indicates the wild type phenotype, and the smaller size band the mutants with gene deletions). Figure S3: Mass spectrum of the main yellow pigment of L. enzymogenes OH11 and light absorption spectra (400-500 nm) of the pigments extracted from various strains. A. wild type OH11; B. ΔORF17; C. ΔORF6; D. ΔORF10; E. ΔORF13; F. xanthomonadins from Xanthomonas campestris pv. campestris. Figure S4: Sequence analysis of ORF16. A. Superposition between ORF16 (white, 275 residues) and the N-terminal region of chain-length factor of actinorhodin PKS, Act_CLF (purple, 415 residues, pdb 1TQY, structure of the KS/CLF heterodimer) [52]. The three green boxes indicate the location of the α3 helix, the loop between α3 and α4 helices, and the α6 helix. The serine residue (S120 for L. enzymogenes ORF16 and S128 for Act_CLF) that is conserved in all sequences is located in the loop between α3 and α4 helices, and the glutamine residue (Q166 for L. enzymogenes ORF16 and Q161 for Act_CLF) that is conserved in all ORF16 homologs is located in the α6 helix. B. Multiple sequence align [file pone.0066633.s001.doc]

**Supporting Information**

**Table S1** Primers used in this study.

OPAC1 5′- ACGAATTCAACACCAGCGAGCAGTTGTT-3′ (*EcoR*I underlined)

OPAC2 5′-CCAAGCTTGTGCTGTTCGGCTTCAATTC-3′ (*Hin*dIII underlined)

OPAC3 5′-AAAAGCTTGCTGCCGCACAGCGTGTCGA-3′ (*Hin*dIII underlined)

OPAC4 5′-AATCTAGAATGCCGCCCCTGGCGATCCG-3′ (*Xba*I underlined)

Cm-1 5′-GGCGCGCCTACCTGTGACGG-3′

Cm-2 5′-CTTACGCCCCGCCCTGCCAC-3′

ORF1F1 5′-CCCGGATCCGCCACGGCCCCAGCCAGCAC-3′ (*Bam*HI underlined)

ORF1R1 5′-CCCAGATCTCATCGCGCGCAGGGCGTACT-3′ (*Bgl*II underlined)

ORF1F2 5′-CCCAGATCTGACGCCACCTTCCTGTCCGG-3′ (*Bgl*II underlined)

ORF1R2 5′-CCCAAGCTTTGGCATCGGTATGCTCCTTT-3′ (*Hin*dIII underlined)

ORF5F1 5′-CCCGGTACCGTAACCGCTATCGCAAGGGG-3′ (*Kpn*I was underlined)

ORF5R1 5′-CCCTCTAGACGGGTCGTCGTGATGCGGAG-3′ (*Xba*I was underlined)

ORF5F2 5′-CCCTCTAGACGGCACGAGTTGCTGGTCGA-3′ (*Xba*I underlined)

ORF5R2 5′-CCCAAGCTTAGCGCATCAACGGCCTGAAC-3′ (*Hin*dIII underlined)

ORF6F1 5′-CCCGGATCCCGAGGGCCGCAAGGGCCGTC-3′ (*Bam*HI underlined)

ORF6R1 5′-CCCAGATCTGCGTTCGGCGGGGCTTTGTT-3′ (*Bgl*II underlined)

ORF6F2 5′-CCCAGATCTCAGCACCGCGCCGCCTGAGC-3′ (*Bgl*II underlined)

ORF6R2 5′-CCCAAGCTTCCATCGTCCACGCCCACAGC-3′ (*Hin*dIII underlined)

ORF10F1 5′-CCCAAGCTTGCAGCACCACGCCCGCAGCG-3′ (*Hin*dIII underlined)

ORF10R1 5′-CCCAGATCTGGCGTCGGGGCGGCGGTATT-3′ (*Bgl*II underlined)

ORF10F2 5′-CCCAGATCTGGCGAACAACAGCGCACCCT-3′ (*Bgl*II underlined)

ORF10R2 5′-CCCGGATCCGAGCGTGGGCGAGAGCAGGT-3′ (*Bam*HI underlined)

ORF13F1 5′-CCCGGATCCATCCCGCCCATCCGCTGCGC-3′ (*Bam*HI underlined)

ORF13R1 5′-CCCAGATCTCGTGTCCGCTGCGCTCATCG-3′ (*Bgl*II underlined)

ORF13F2 5′-CCCAGATCTATCGGGGTCAACGGCGGCAT-3′ (*Bgl*II underlined)

ORF13R2 5′-CCCAAGCTTCGCCGCCGCCGCTGAAATGG-3′ (*Hin*dIII underlined)

ORF16-V-F 5′-CGCGCCTTCGACCACTTC3′

ORF16-V-R 5′-ACCATGATTACGCCAAGC3′

ORF18F1 5′-CCCGGATCCCCAGCAGCAGAAGAAGACCC-3′ (*Bam*HI underlined)

ORF18R1 5′-CCCAGATCTAGGGGGCGGAACGGAACGGG-3′ (*Bgl*II underlined)

ORF18F2 5′-CCCAGATCTCGGGCGATGGGGCTGGAGCC-3′ (*Bgl*II underlined)

ORF18R2 5′-CCCAAGCTTGCGGCGGGAAGTCGAGCATC-3′ (*Hin*dIII underlined)

Q/S-up-forw 5′-CCGCTCGAGGAAGAGGCGTCAGTCATGGTCC-3′

Q-up-rev 5′-GGCGCTGGCGGCGCTGGCGGCCGGCGAA-3′

Q-down-forw 5′-CGGCCGCCAGCGCCGCCAGCGCCTCGAT-3′

Q/S-down-rev 5′-CGGGATCCGCGGCCTGCCCGACTGGAAC-3′ (*Bam*HI underlined)

S-up-rev 5′-GCGCGCGATTGCGCCGACCCGCTTCCAC-3′

S-down-forw 5′-AGCGGGTCGGCGCAATCGCGCGCGGATC-3′

**A.**


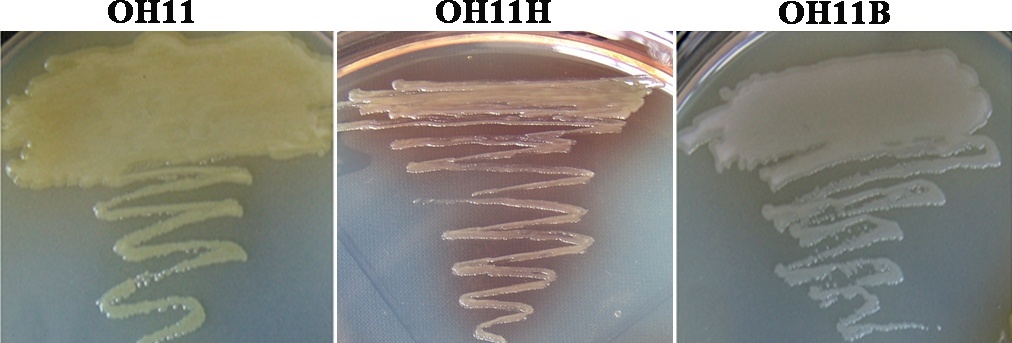

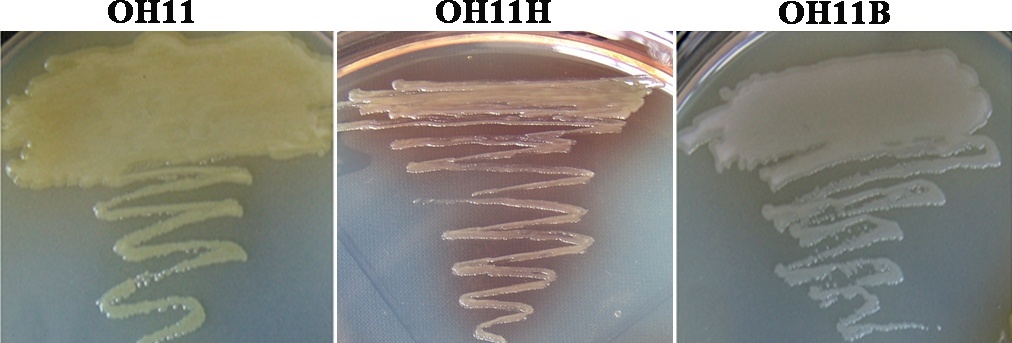


**B.**


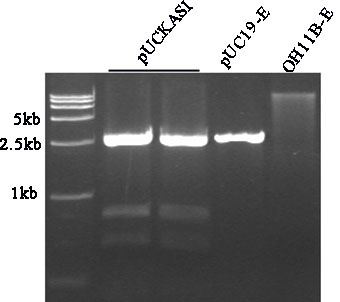

**Figure S1** Identification of the yellow pigment deficient *L. enzymogenes* OH11 mutant through transposon random mutagenesis. A. phenotype of colonies. OH11, the wild type; OH11B, the “white” mutant. B. Cloning the flanking region of the transposon insertion site in the mutant. The genomic DNA was digested by *Eco*RI and cloned into pUC19. The colonies with chloramphenicol (Cm) resistance were the putative positive hits, which were verified by DNA sequencing. pUCKASI, *Eco*RI-digested plasmid from Cm-resistant colonies; pUC19-E, *Eco*RI-digested vector pUC19; OH11B-E, *Eco*RI-digested genomic DNA of mutant OH11B.

**A**

**
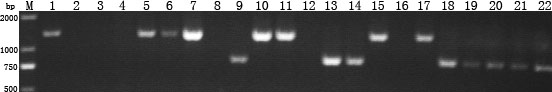
**

**B**

**
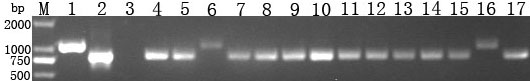
**

**C**

**
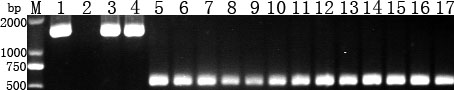
**

**D**

**
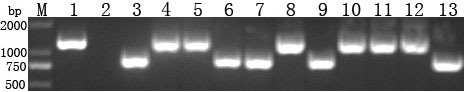
**

**E**

**
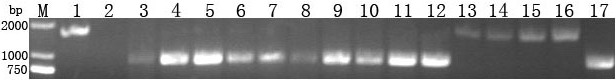
**

**F**

**
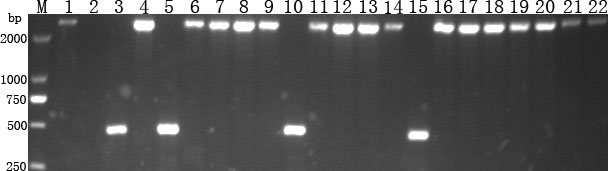
**

**
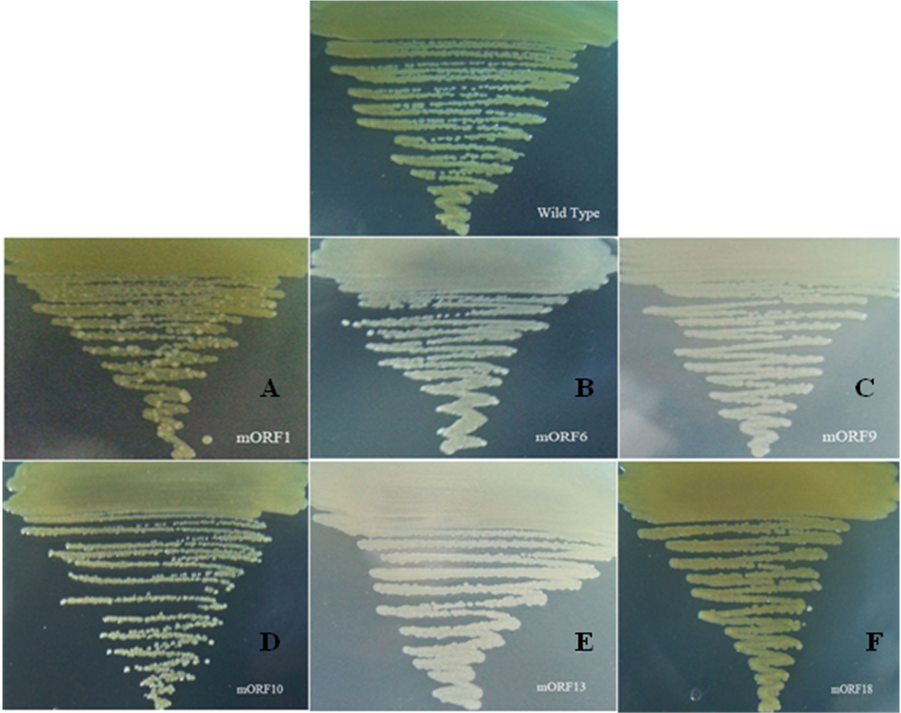
**

**Figure S2** PCR verification of gene deletion mutants and the phenotype of the mutants. A. ∆ORF1; B. ∆ORF6; C. ∆ORF9; D. ∆ORF10; E. ∆ORF13; F. ∆ORF18. M, size markers; lane-1, wild type; the rest lanes, individual colonies that were selected for analysis (the larger size band indicates the wild type phenotype, and the smaller size band the mutants with gene deletions).


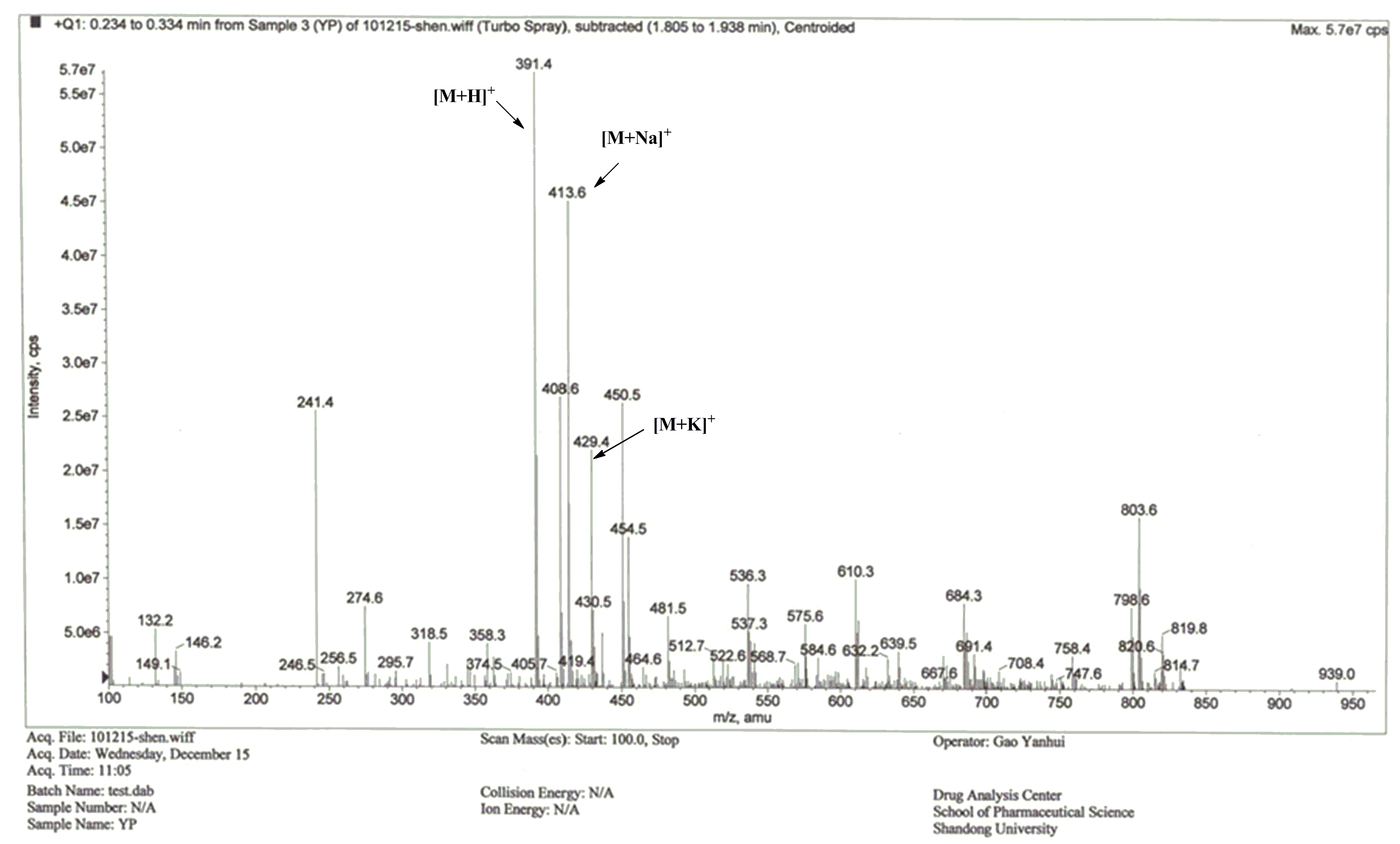


**
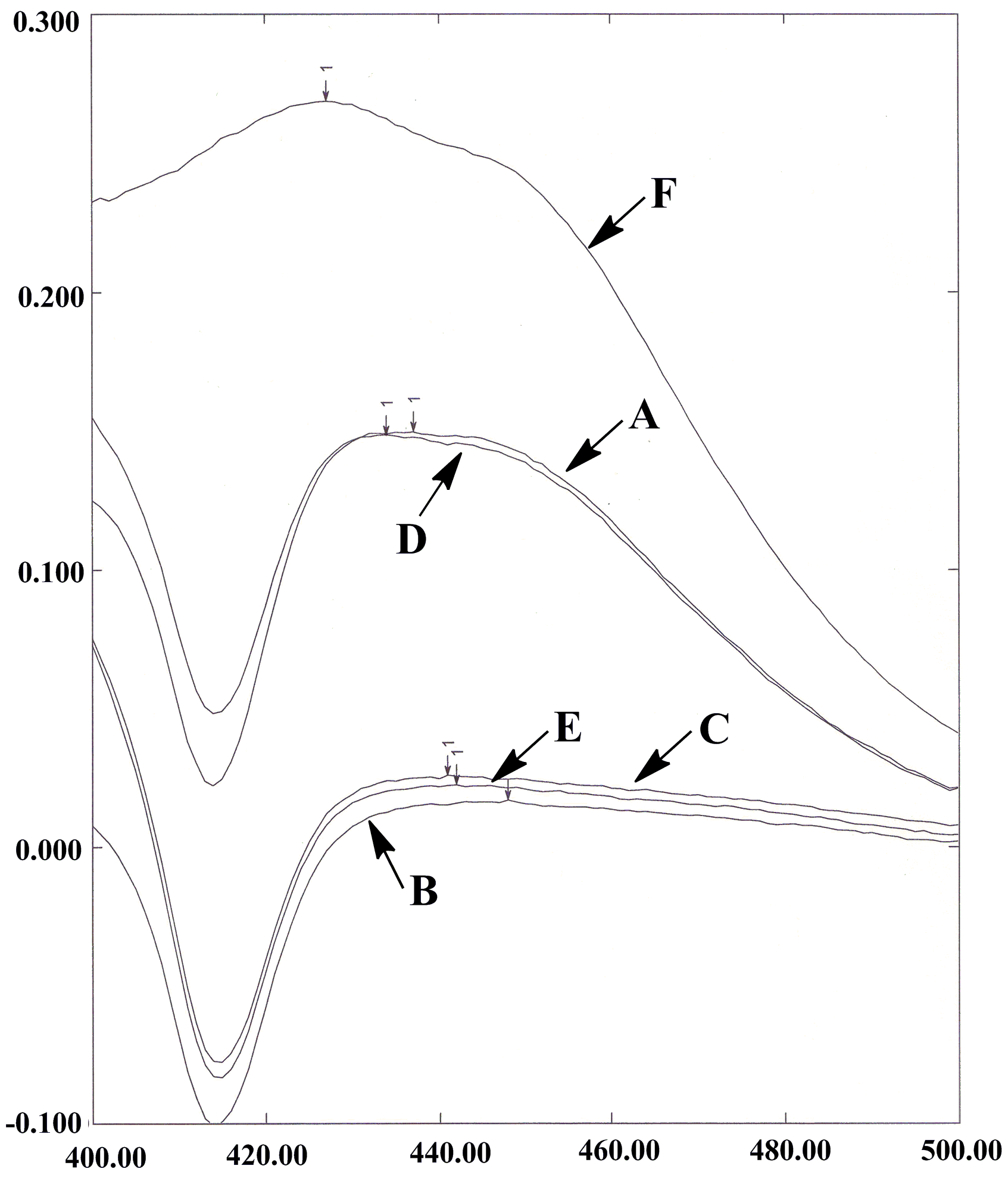
**

**Figure S3** Mass spectrum of the main yellow pigment of *L. enzymogenes* OH11 and light absorption spectra (400-500 nm) of the pigments extracted from various strains. A. wild type OH11; B. ∆ORF17; C. ∆ORF6; D. ∆ORF10; E. ∆ORF13; F. xanthomonadins from *Xanthomonas campestris* pv. *campestris*.

A.


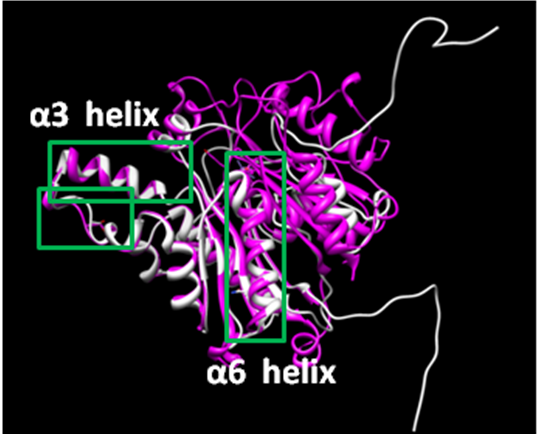

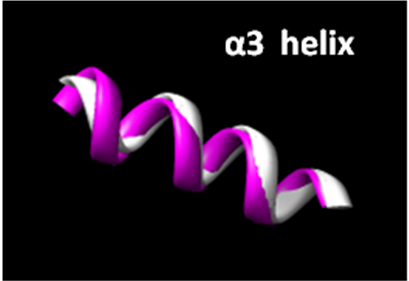


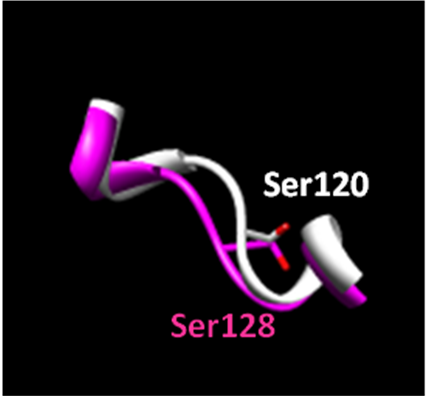

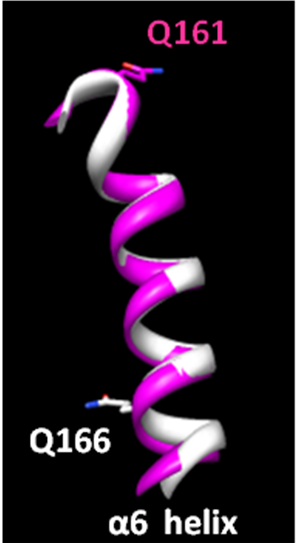


B.

**Figure S4** Sequence analysis of ORF16. A. Superposition between ORF16 (white, 275 residues) and the N-terminal region of chain-length factor of actinorhodin PKS, Act_CLF (purple, 415 residues, pdb 1TQY, structure of the KS/CLF heterodimer). The three green boxes indicate the location of the α3 helix, the loop between α3 and α4 helices, and the α6 helix. The serine residue (S120 for *L. enzymogenes* ORF16 and S128 for Act_CLF) that is conserved in all sequences is located in the loop between α3 and α4 helices, and the glutamine residue (Q166 for *L. enzymogenes* ORF16 and Q161 for Act_CLF) that is conserved in all ORF16 homologs is located in the α6 helix. B. Multiple sequence alignment of selected ORF16 homologs found in GenBank. The alignment was carried out by using Clustal 2.1, and only the sequences around the known active site of known CLF and KS are shown. Tcm_CLF, chain-length factor of tetracenomycin PKS (M80674); Act_CLF and Act_KS, chain-length factor and ketosynthase of actinorhodin PKS (X63449). The glutamine residue conserved in the known CLFs is indicated with a red box, and the active site motif, including the absolutely conserved cysteine residue, of the known KS is underlined. Also indicated by boxes are Q166 and S120, which were subject to mutagenesis in ORF16.
